# Phenoloxidases: catechol oxidase – the temporary employer and laccase – the rising star of vascular plants

**DOI:** 10.1093/hr/uhad102

**Published:** 2023-05-16

**Authors:** Jugou Liao, Xuemei Wei, Keliang Tao, Gang Deng, Jie Shu, Qin Qiao, Gonglin Chen, Zhuo Wei, Meihui Fan, Shah Saud, Shah Fahad, Suiyun Chen

**Affiliations:** School of Ecology and Environmental Sciences, Yunnan University; Biocontrol Engineering Research Center of Crop Diseases & Pests, Yunnan Province, Kunming 650091, China; School of Engineering, Dali University, Dali, Yunnan Province, 671003, China; School of Life Science, Yunnan University, Yunnan Province, Kunming 650091, China; College of Horticulture and Landscape, Yunnan Agricultural University, Yunnan Province, Kunming 650091, China; School of Life Science, Yunnan University, Yunnan Province, Kunming 650091, China; College of Horticulture and Landscape, Yunnan Agricultural University, Yunnan Province, Kunming 650091, China; School of Ecology and Environmental Sciences, Yunnan University; Biocontrol Engineering Research Center of Crop Diseases & Pests, Yunnan Province, Kunming 650091, China; School of Ecology and Environmental Sciences, Yunnan University; Biocontrol Engineering Research Center of Crop Diseases & Pests, Yunnan Province, Kunming 650091, China; School of Ecology and Environmental Sciences, Yunnan University; Biocontrol Engineering Research Center of Crop Diseases & Pests, Yunnan Province, Kunming 650091, China; College of Life Science, Linyi University, Linyi, Shandong 276000, China; Department of Agronomy, Abdul Wali Khan University Mardan, Khyber Pakhtunkhwa 23200, Pakistan; School of Ecology and Environmental Sciences, Yunnan University; Biocontrol Engineering Research Center of Crop Diseases & Pests, Yunnan Province, Kunming 650091, China

## Abstract

Phenolics are vital for the adaptation of plants to terrestrial habitats and for species diversity. Phenoloxidases (catechol oxidases, COs, and laccases, LACs) are responsible for the oxidation and polymerization of phenolics. However, their origin, evolution, and differential roles during plant development and land colonization are unclear. We performed the phylogeny, domain, amino acids, compositional biases, and intron analyses to clarify the origin and evolution of COs and LACs, and analysed the structure, selective pressure, and chloroplast targeting to understand the species-dependent distribution of COs. We found that Streptophyta COs were not homologous to the Chlorophyta tyrosinases (TYRs), and might have been acquired by horizontal gene transfer from bacteria. COs expanded in bryophytes. Structural-functionality and selective pressure were partially responsible for the species-dependent retention of COs in embryophytes. LACs emerged in Zygnemaphyceae, having evolved from ascorbate oxidases (AAOs), and prevailed in the vascular plants and strongly expanded in seed plants. COs and LACs coevolved with the phenolic metabolism pathway genes. These results suggested that TYRs and AAOs were the first-stage phenoloxidases in Chlorophyta. COs might be the second key for the early land colonization. LACs were the third one (dominating in the vascular plants) and might be advantageous for diversified phenol substrates and the erect growth of plants. This work provided new insights into how phenoloxidases evolved and were devoted to plant evolution.

## Introduction

During the colonization of terrestrial environments, plants were challenged by desiccation, fluctuating temperatures, osmotic pressure, UV irradiation, and pathogen infection [1, 2]. The successful land adaptation was achieved largely by protection from the ‘UV light screens’ of phenolic compounds [3]. Phenolics, especially flavonoids, also function in attracting pollinators for plant reproduction, and in regulating plant hormonal activity [4, 5]. Phenolic compounds are derived from the shikimate, phenylpropanoid and flavonoids pathway [6]. Once synthesized and transported to specific cellular compartments, phenolic monomers are polymerized by phenoloxidases [6].

**Figure 1:**
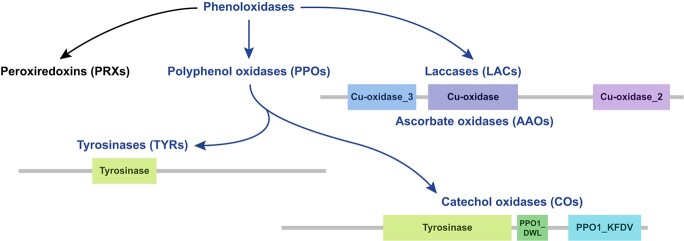
The phenoloxidases and their domain structure. Polyphenol oxidases (PPOs) generally refer to tyrosinases (TYRs) and catechol oxidases (COs). Both have the N-terminus tyrosinase domain. The laccases (LACs) and ascorbate oxidases (AAOs) share the same PFAM (Protein Families) domains. This study focused on the enzymes in blue font.

Phenoloxidases are usually used to describe phylogenetically unrelated three families of enzymes, including peroxiredoxins (PRXs), polyphenol oxidases (PPOs) and laccases (LACs) [6] (Fig. 1). These enzymes are grouped together by their common capacity to oxidize substrates containing a phenolic ring [6]. Phenoloxidases fulfill two functions: removing excess free oxygen radicals [7], and catalyzing the oxidation and polymerization of various polyphenolic compounds [6]. Therefore, phenoloxidases are essential not only for fundamental physiological processes, but also for supporting plants to adapt to, and thrive in terrestrial habitats [6].

Tyrosinases (TYRs, EC 1.14.18.1) and catechol oxidases (COs, EC 1.10.3.1) are commonly referred to as PPOs based on their substrate specificity and structure [8, 9] (Fig. 1). TYRs and COs share the N-terminus tyrosinase domain (PF00264, harboring the enzymatic center) [10]. Mature COs contain an additional short linker region of the PPO1_DWL domain (PF12142) [11], and a C-terminus PPO1_KFDV domain (PF12143) covering the active site of the enzyme [12] (Fig. 1).

TYRs are the major enzymes responsible for the formation of melanin pigment, and are found throughout the animal kingdom [13]. TYRs catalyze the hydroxylating tyrosine to DOPA (3,4-dihydroxyphenylalanine), the oxidation of DOPA to DOPA-quinone, and o-hydroxylation of monophenols [11]. In plants, PPOs usually refer to the COs. COs can hydroxylate their potential natural monophenol substrates and oxidize o-diphenols [14]. Their substrates are usually species-specific [12, 15, 16].

Laccases (LACs, EC 1.10.3.2) and ascorbate oxidases (AAOs, EC 1.10.3.3) are members of the multi-copper-oxidase family (MCOs) and share the same PFAM (Protein Families) domains [17] (Fig. 1). LACs are the largest subgroup of MCOs, present in animals, plants, bacteria, and fungi [18]. In plant species, they occur as large multigenic families [19]. LACs catalyze successive one-electron oxidations of a wide variety of organic and inorganic substrates, such as mono-, di-, and polyphenols, polyamines, and certain inorganic compounds [17]. AAOs are apoplastic enzymes catalyzing the oxidation of ascorbate to monodehydroascorbate [20].

The overlapping oxidation activity against phenols was found among LACs and COs [21]. LACs can oxidize most types of phenolics, with much more remarkable catalytic abilities and broader substrate specificity in contrast to COs [6, 22]. However, as widely distributed and functionally pivotal phenoloxidases, how LACs and COs differentially contributed to the land colonization, and evolution of plants, remains unclear. Additionally, the origin and evolution of Streptophyta COs and LACs are worthy of further investigation. Our analysis showed that Streptophyta COs were not homologous to the Chlorophyta TYRs, and might have been introduced by horizontal gene transfer (HGT) from bacteria. COs expanded in bryophytes, and were species-dependently retained in embryophytes. The LACs evolved from the AAOs and prevailed in the vascular plants. These findings would greatly facilitate our understanding of the specialized and divergent functions of COs and LACs in plant evolution, and are likely to shed light on their gene functions.

## Results

### The Streptophyta COs were not homologous to the Chlorophyta TYRs

**Figure 2:**
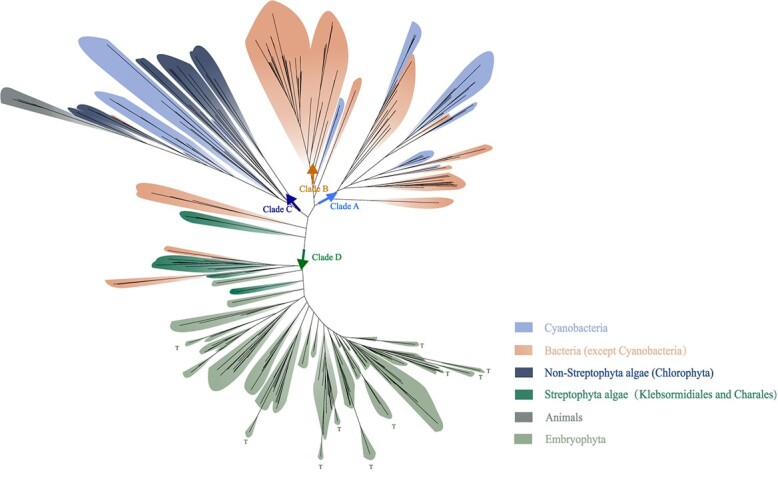
Phylogenetic analysis shows Streptophyta catechol oxidases (COs) are not homologous to the Chlorophyta tyrosinases. Sequences of three conserved domains from bacteria, cyanobacteria, chlorophytes, and animals were extracted to build the maximum likelihood tree using IQ-TREE in PhyloSuite. The best model was VT + G4 detected by the BIC criterion in ModelFinder, and 20 000 replicates of ultrafast bootstraps were computed. T refers to the embryophytes COs with only the tyrosinase domain.

Given that TYRs and COs share the N-terminus tyrosinase domain, we wondered if COs originated from TYRs and subsequently developed the additional two domains. To explore the origin and evolution of plant COs, we computed a maximum likelihood phylogeny from a multiple sequence alignment of the three conserved domains in PPOs from plants, animals, and bacteria (including cyanobacteria). The phylogenetic tree had four clades. The A and B clades included most of the cyanobacterial and bacterial PPOs (Fig. 2). The C clade comprised PPOs from animals, cyanobacteria, and chlorophytes with only the tyrosinase domain (Figs 1 and 2; Fig. S1a, see online supplementary material). Therefore, the PPOs of chlorophytes were TRYs with a relatively close genetic alignment to those of animals and cyanobacteria.

**Figure 3:**
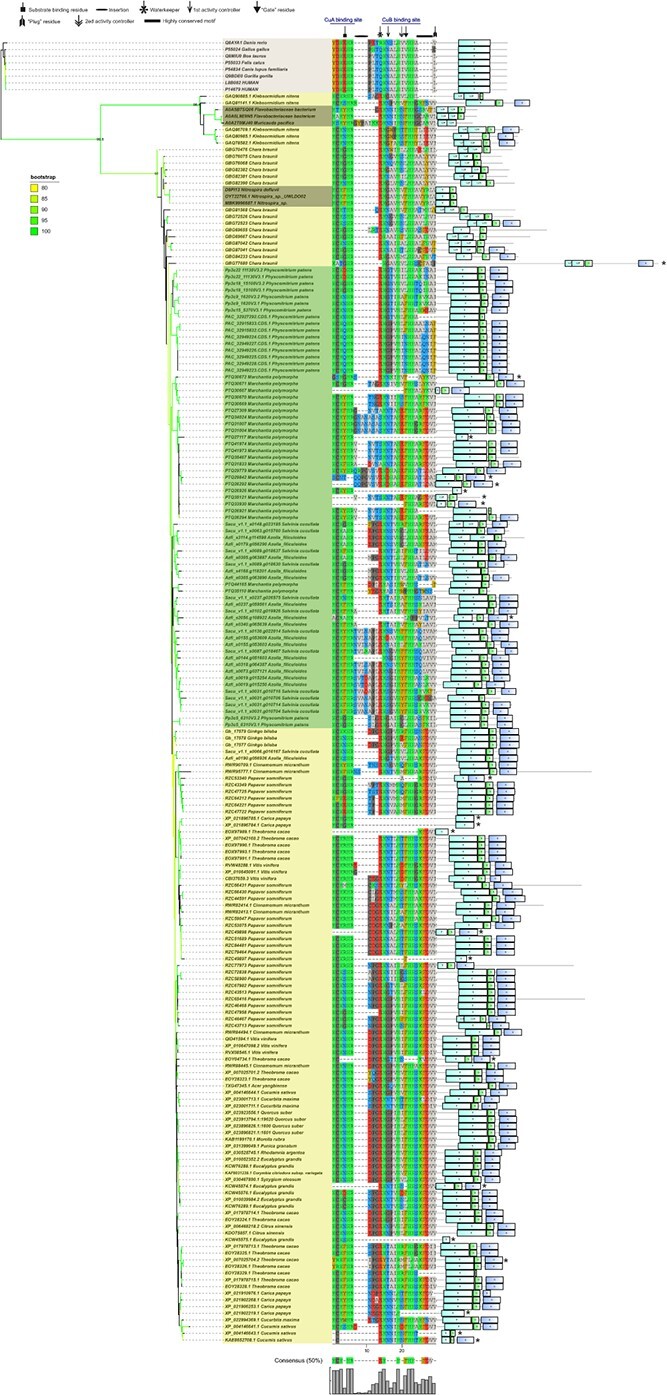
Phylogenetic, amino acids, and domain analyses support the bacterial origin of Streptophyta catechol oxidases (COs). We computed a maximum likelihood phylogeny from the three conserved domains of the ancestral bacteria and Streptophyta COs, rooted by the animal tyrosinases. The nodes with bootstrap values >80% were colored in the figure, and the bootstrap legend is in the top-left corner. The main nodes were also assigned with Shimodaira–Hasegawa-like approximate likelihood-ratio test (SH-aLRT) value (numbers in the nodes). In the middle we show the residues around the substrate-binding pockets. The asterisks refer to structural non-functional COs (with any of the six His residues absent). The domains were predicted using PfamScan and are showed on the right.

The PPOs of the Streptophyta algae and embryophytes were all included in the D clade (Fig. 1), where five PPOs of the Nitrospiraceae and Flavobacteriaceae were also found. This finding suggested that there might have been a horizontal gene transfer (HGT) from these bacteria to the Streptophyta algae. The PPOs of *Klebsormidium nitens* and *Chara braunii* were genetically close to those of Flavobacteriaceae and Nitrospiraceae*,* respectively (Figs 2 and 3). The amino acids around the substrate-binding pockets of streptophyte algae PPOs were different from those of chlorophytes TRYs (Fig. S2, see online supplementary material). Notably, a few PPO members of the embryophytes with only the first tyrosinase domain were also part of clade D (Figs 2 and 3), and their 3-D structure was more like that of streptophyte PPOs than chlorophytes TRYs (Fig. S1a–f, see online supplementary material). These data revealed that the streptophyte PPOs with only the tyrosinase domain had the same origin with the Streptophyta COs, and probably had lost the second and third domain during evolution.

Therefore, the PPOs in chlorophytes were TYRs, while those in the Streptophyta were all COs. The Streptophyta COs did not originate from the Chlorophyta TYRs, and were gained probably by a HGT from bacteria. These results agreed with the notion that some representatives of green algae had PPO proteins containing only TYR domains [23], while being inconsistent with a suggestion of the absence of COs in streptophyte algae, and with PPOs becoming important in bryophytes [24]. The PPO activity was reported in chlorophytes and charophytes [6, 25], and the activity in chlorophytes and charophytes was due to TYRs and COs, respectively, according to our data.

### COs of Streptophyta algae were homologous to those of bacteria

To further verify the homology of streptophyte COs to those of bacteria, we computed a maximum likelihood phylogeny of the three conserved domains of the plant and the ancestral bacterial COs, rooted by the TYRs of animals. The phylogenetic analysis showed that the COs of Nitrospiraceae, *C. braunii* and embryophytes grouped into one clade with strong phylogenetic signals (bootstrap support and SH-aLRT value >90%). The ‘core’-Streptophyta (Zygnematophyceae, Coleochaetophyceae, and Charophyceae, ZCC grade) [26] might gain COs by a single HGT from ancestral bacteria (Fig. 3). The domain structure analysis showed that most of the COs in streptophyte algae (13 out of 16 in *C. braunii*, and two out of five in *K. nitens*) had only the tyrosinase and PPO1_DWL domains, like those of Nitrospiraceae and Flavobacteriaceae, supporting their homology (Fig. 3; Fig. S1b–d, see online supplementary material). The PPO1_KFDV domain was detected in 3 of 15 COs in *C. braunii* (Fig. 3; Fig. S1g, see online supplementary material) and might have arisen after the transfer of COs from bacteria. This PPO1_KFDV domain was contained in 20 of 32 PPOs in *Physcomitrium patens*, and was more prevalent in ferns and seed plants (Fig. 3; Fig. S1h and i, see online supplementary material). Furthermore, the highly conserved KFDV motif in this newly formed domain was not found in the COs of charophytes and bryophytes, and was detected in two COs of ferns, but was universal in angiosperms (Fig. 3). These findings indicated that the PPO1_KFDV domain and the conserved KFDV motif evolved gradually in Streptophyta.

Amino acids neighboring the six conserved His residues of CuA and CuB (especially these around CuB) are key for the substrate-binding and selectivity [12, 27, 28]. We analysed these amino acids to test the origin and evolution of Streptophyta COs. The gatekeeper residue is a conserved phenylalanine (F) in plant COs [10, 27]; however, our analysis showed that this F residue was frequently replaced by leucine (L) in most COs of *C. braunii*, and by asparagine (N) or L in *P. patens* (Fig. 3). The F residue prevailed in ferns and dominated in the COs of seed plants (Fig. 3). As this gatekeeper residue was F in COs of Nitrospiraceae, Flavobacteriaceae, and in at least one COs of our analysed Streptophyta species (Fig. 3), we speculated that this residue might have mutated after transfer from the bacteria and been positively selected and retained in the vascular plants.

The amino acid residue at position HB2 + 1 acts as a second activity controller and determines substrate preference depending on its charge [29]. This residue was not conserved among Streptophyta. Specific amino acids predominated in some phylogroups, such as isoleucine (I) in *P. patens*, a negatively charged glutamic acid (E) in *Marchantia polymorpha*, and tyrosine (Y) in ferns (Fig. 3). These taxon-specific amino acids in the second substrate selector agreed with the reported species-dependent substrate preferences of COs [15, 16, 30]. The first activity controller HB1 + 1 residue was more conserved and was mainly glycine (G) or asparagine (N) in most COs, similar to that in their bacterial ancestor (Fig. 3). The waterkeeper residue Glu (E) [27] was conserved among almost all characterized Streptophyta COs, and their bacterial ancestor (Fig. 3) but was hardly found in the Chlorophyta TYRs (Fig. S2, see online supplementary material).

Compositional biases and introns were further analysed to verify the homology of streptophyte COs to those of bacteria. The compositional biases of streptophyte algae were more similar to that of Nitrospiraceae (Fig. S3, see online supplementary material), further supporting their homology. Furthermore, introns were found rarely in COs of *C. braunii*, while the number of genes with introns increased in *M. polymorpha* and seed plants (Fig. S4, see online supplementary material). The results of phylogeny, domain structure, key residues, the 3-D structures, compositional biases, and introns collectively supported the homology of Streptophyta COs to that of bacteria.

### The LACs emerged in Zygnemaphyceae and originated from the AAOs

**Figure 4:**
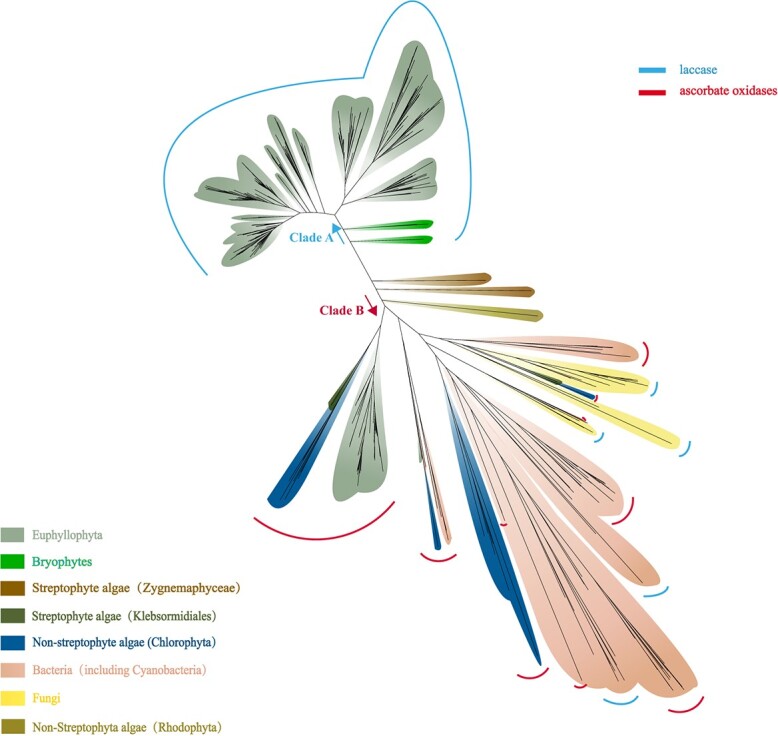
The gradual evolution of plant laccases (LACs) from the ascorbate oxidases (AAOs). Conserved domain sequences from AAOs and LACs of bacteria, cyanobacteria, algae (Chlorophyta, Klebsormidiales, and Rhodophyta), and fungi were extracted to build the maximum likelihood tree using IQ-TREE in PhyloSuite. The best model was LG + I + G4 + F detected by the BIC criterion in ModelFinder, and 10 000 replicates of ultrafast bootstraps were computed. The red and blue arcs refer to the AAOs and LACs, respectively.

LACs are another class of phenoloxidases sharing some substrates with the COs [21]; hence, the origin and evolution of LACs were also analysed to explore their differential contribution to land colonization. LACs and AAOs share the same PFAM domains, and it was speculated that the AAOs might be the ancestral homologs of LACs [6]. This hypothesis was verified in the present study. The LACs and AAOs of distantly related plant lineages and those of bacteria and fungi were obtained. A maximum likelihood phylogenetic tree was generated from a multiple sequence alignment of three Cu-oxidase domains of the LACs and AAOs. The analysis separated the LACs and AAOs into two clades. Clade A included the LACs and AAOs of bacteria (including cyanobacteria), and the AAOs of red algae, chlorophytes, charophytes (*K. nitens*), and land plants (Fig. 4). Clade B included the land plant LACs. Notably, the identified earliest LACs of *M. polymorpha* and *P. patens* [6] were between clades A and B (Fig. 4).

**Figure 5:**
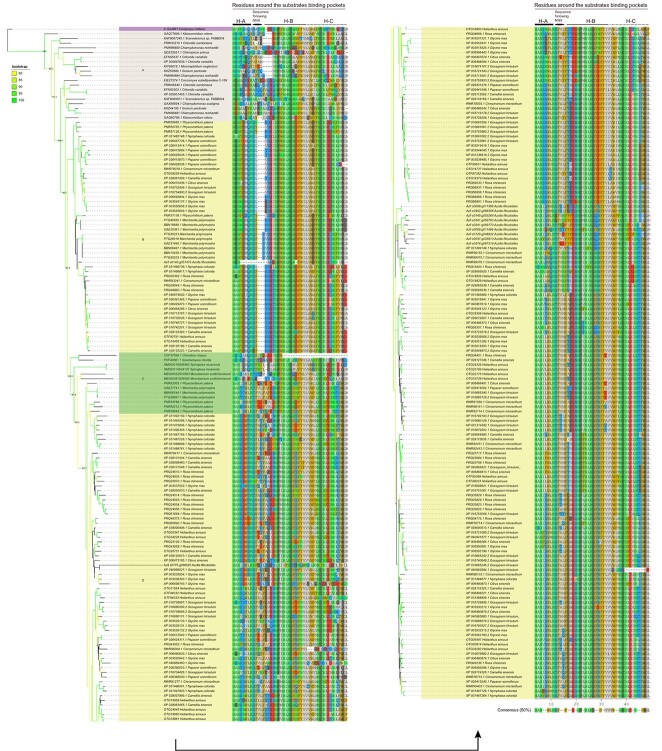
Root tree and key residue analysis show that the earliest plant laccases occurred in the Zygnemaphyceae and Rhodophyta. We computed a maximum likelihood phylogeny from the three conserved domains of plant ascorbate oxidases (AAOs) and laccases (LACs), rooted by the fungi AAOs (G3J8R1). The A and B clades include the AAOs of algae (chlorophytes and Klebsormidiales) and land plants, respectively. C clade comprises the earliest LACs of Rhodophyta, Zygnemaphyceae and bryophytes. The LACs of vascular plants are grouped into clade D. The nodes with bootstrap values >80% were colored in the figure, and the bootstrap legend is in the top-left corner. The main nodes were also assigned with SH-aLRT (values in the nodes). Residues around the substrate-binding pockets are characterized by three histidine regions denoted as H-A, H-B, and H-C. Residues following the NNX sequences are also near the pockets.

To analyse the origin and evolution of LACs further, a phylogenetic tree of three conserved domains of plant LACs and AAOs was generated, rooted by the AAOs of fungi. The phylogenetic analysis showed that the land plant AAOs (clade B) formed a sister group with the streptophyte LACs (clade C and D, Fig. 5). The AAOs in chlorophytes (clade A) had evolved into two directions: the land plant AAOs and the streptophyte LACs (Fig. 5). Therefore, the Streptophyta LACs originated from the Chlorophyta AAOs.

Four sequences of *Mesotaenium endlicherianum* and *Spirogloea muscicola* (Zygnemaphyceae) were between the identified earliest LACs of bryophytes and the AAOs (Fig. 4). It remained unclear whether these four sequences were LACs or AAOs, as they were not annotated in the public databases. In the rooted Fig. 5, these unidentified sequences were classified into the clade C of streptophyte LACs, revealing they are genetically close to the LACs, and might be the earliest streptophyte LACs.

Laccases contain four copper ions: type 1 (T1), type 2 (T2), and double type 3 (T3α, T3β). The amino acids surrounding the substrate-binding pocket near the T1 Cu center of domain 3 influence the substrate selectivity and catalytic properties [31–33]. We further extracted and compared these amino acid residues to explore the origin and evolution of LACs; the amino acids of four potential LACs of Zygnemaphyceae were more like those of the identified LACs in bryophytes (Fig. 5), further supporting the notion they are the plant LACs. Additionally, CDF37599.1 and PXF40661.1 (Rhodophyta) were annotated as AAOs in Uniport database; however, they clustered to the clade C of streptophyte LACs (Fig. 5). Similarly, laccase-like enzyme activity was described in red algae and soil green algae *Tetracystis aeria* [34]. Therefore, the bona fide LACs emerged in the Rhodophyta and Zygnemaphyceae, which agreed with the prediction that bona fide LACs might have originated in multicellular green algae or early land plants [6], but was inconsistent with LACs having evolved first in bryophytes [22].

The four amino acids following a conserved NNX motif near the substrate-binding pockets varied frequently, especially in the AAOs of chlorophytes (Fig. 5), revealing their diverse substrate selectivity. For the land plant AAOs, these four residues were frequently lost, and other residues surrounding the substrate-binding pockets were more conserved (Fig. 5). This indicated that the land plant AAOs might be specialized in substrate recognition and catalysis. The amino acids around substrate-binding pockets of LACs were more polymorphic than those of land plant AAOs, especially the sequences following the NNX motif (Fig. 5), agreeing with their capacity to oxidize a wide range of substrates [6].

In summary, the AAOs first appeared in chlorophytes and Klebsormidiales, and evolved into the more specific land plant AAOs and the diverse streptophyte LACs. The LACs evolved slowly and gradually, and emerged in streptophyte algae (Zygnemaphyceae) and two species of Rhodophyta.

### The expansion and contraction of COs and LACs, and their coevolution with the phenolic metabolism genes

**Figure 6:**
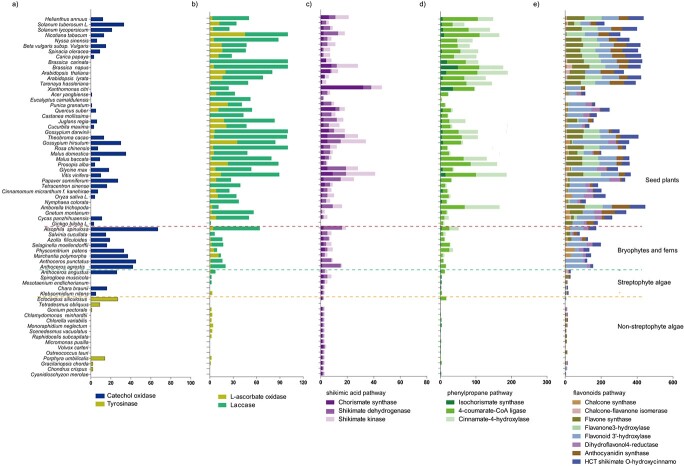
The coevolution of laccases (LACs) and catechol oxidases (COs) with the phenolic deriving pathway genes. The **a** column refers to the gene number of COs and tyrosinases (TYRs). The **b** column is the number of ascorbate oxidases (AAOs) and laccases (LACs). The **c**, **d**, and e columns represent the number of genes in the shikimic acid, phenylpropane, and flavonoids pathways, respectively.

**Figure 7:**
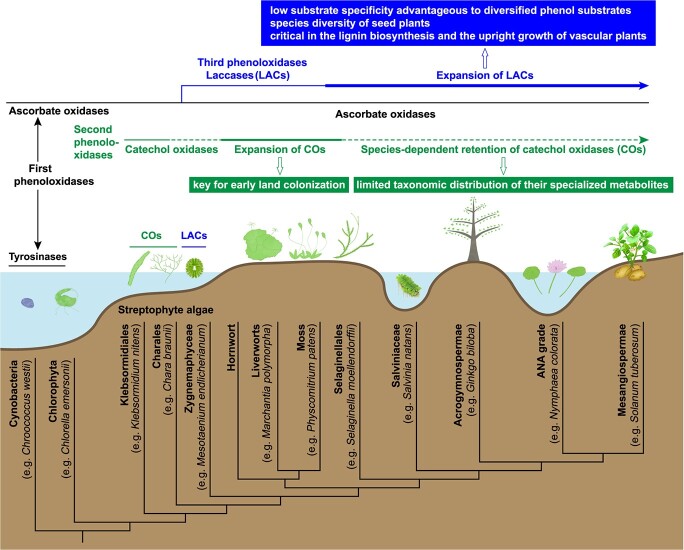
The first, second, and third stage phenoloxidases during plant evolution. The bold blue and green lines represent the expansion of laccases and catechol oxidases (COs), respectively. Dotted lines mean the species-dependent retention of COs among the species. Note: As there were aquatic, semi-terrestrial, and terrestrial species in the groups of chlorophytes, streptophyte algae, bryophytes, ferns, and seed plants [36], this figure represents only the habitats of the exampled species.

To explore the distinctive role of LACs and PPOs in land adaption and plant evolution, we analysed their gene family expansion and contraction in plants. Only TYRs and AAOs were detected in chlorophytes and were defined as the first-stage phenoloxidases (Figs 6 and 7). The COs occurred in the Klebsormidiales and Charales (5–16), and the gene family expanded strongly in bryophytes (30–58) and ferns ( 15-19 in Salviniaceae, and 67 in *Alsophila spinulosa*) (Figs 6 and 7). COs were accordingly referred as the second-stage phenoloxidases (Fig. 7). The number of COs family members varied from 0 to 35 in angiosperms (Fig. 6). COs were frequently absent from the genome of seed plants, such as in *Gnetum montanum*, the ANA grade, and in the Brassicales except for *Carica papaya* (Figs 6 and 7). The COs family also contracted in the NCBI-contained genomes of Sapindales and Myrtales, with only one member found in the genomes of *Punica granatum* and *Acer yangbiense*, and none was detected in *Eucalyptus camaldulensis* (Fig. 6). Of the 35 embryogenic species we analyzed, only *Gossypium hirsutum* (30), *Solanum tuberosum* L. (33) and *Malus domestica* (35) contained no less than 30 COs in their genome (Fig. 6, Table 1).

The number of AAOs was more than LACs in *P. patens* and *M. polymorpha* (Figs 6 and 7). However, the trend in hornworts and ferns was opposite (Fig. 6). The LACs family expanded greatly in the seed plants (Figs 6 and 7), and there were more than 30 LACs in 72.9% of the 37 analysed species, and 43.2% of these species had more than 50 LACs (Fig. 6). Collectively, these data showed that the LACs were more dominant in seed plants and were third-stage phenoloxidases (Fig. 7).

Polyphenols (phenolic acids and flavonoids) are derived mainly from the shikimic acid, phenylpropane and flavonoids pathways. The PPOs and LACs oxidize and polymerize these metabolites [16, 19, 22]. To better understand the coevolution of phenoloxidases and the polyphenols synthesis genes, we further analysed the number of representative genes in the above pathways. When the first-stage phenoloxidases (AAOs and TYRs) arose in non-streptophyte algae (Fig. 7), the genes of the shikimic acid and flavonoids pathway (probably the earliest phenolics producers in plants) also appeared (Fig. 6). AAOs and TYRs might be responsible for the oxidation and polymerization of the phenolic products in these species. A small number of genes associated with the phenylpropanoid biosynthesis were also found in the non-streptophyte and streptophyte algae we analysed, agreeing with the notion that the phenylpropanoid biosynthetic genes were present in streptophyte algae[35] [36], and even in chlorophytes [5, 36–38].

The number of genes in the shikimic acid and flavonoids pathway increased, when COs (in Klebsormidiales and Charales) and LACs (in Zygnemaphyceae) emerged in streptophyte algae. In bryophytes and ferns, the expansion of COs and the abundance of LACs were accompanied by a larger number of gene family members in the shikimic acid, flavonoids, and phenylpropane pathway genes (Fig. 6). Notably, the gene family of LACs and the shikimic acid, phenylpropane and flavonoids pathways expanded strongly in seed plants (Fig. 6). These data agreed with the flavonoid compounds occurring commonly during the water-to-land transition, and increasing to attain the largest variety in seed plants [3, 39]. Therefore, COs and LACs coevolved with genes involved in the biosynthesis of their substrates. This coevolution is vital for the UV-B protection, resistance to biotic and abiotic stresses, the erect growth of vascular plants, and visual signals for attracting pollinators, underpinning the emergence of huge plant diversity [5, 40].

**Table 1:** The selective pressure, enzymatic center non-functionality and chloroplast targeting of catechol oxidases (COs)

Species	Non-functionality ratio	Purifying selection (Ka/Ks < 1)	Mean	Positive selection (Ka/Ks > 1)	Mean	Neutral evolution (Ka/Ks = 1)	Potential chloroplast-localized COs
*Klebsormidium nitens*	0 of 5	1 of 2	0.91	1 of 2	1.32		1 of 5
*Chara braunii*	1 of 15^FP^	1 of 11	0.92	10 of 11^FP^	1.31		1 of 15
*Physcomitrium patens*	2 of 33	9 of 19	0.62	1 of 19	1.54	9 of 19	3 of 33
*Marchantia ploymorpha*	23 of 77^NN^	90 of 95^NN^	0.22	2 of 95	1.19	3 of 95	15 of 77
*Azolla filiculoides*	3 of 18 ^FP^	4 of 16	0.75	8 of 16^FP^	1.28	4 of 16	6 of 18
*Cycas panzhihuaensis*	4 of 11	2 of 11	0.61	1 of 11	1.17	8 of 11	7 of 11
*Cinnamomum micranthum*	1 of 9 ^FP^			3 of 3 ^FP^	1.62		5 of 9
*Oryza sativa* Japonica	14 of 21	2 of 9	0.77	4 of 9	1.45	3 of 9	6 of 21
*Nicotiana tabacum*	0 of 14 ^FP^			23 of 23^FP^	1.67		12 of 14
*Solanum tuberosum Vitis vinifera*	9 of 33 4 of 16	33 of 63 1 of 10	0.56 0.85	18 of 63 6 of 10	1.65 1.29	12 of 63 3 of 10	21 of 33 11 of 16
*Nyssa sinensis*	3 of 7	2 of 3	0.42	1 of 3	2.49		4 of 7
*Glycine max*	8 of 22^NN^	8 of 12^NN^	0.56	2 of 12	1.18	2 of 12	15 of 22
*Malus domestica Cucurbita maxima*	6 of 35 0 of 3	52 of 85 1 of 1	0.53 0.93	5 of 85	1.21	28/85	20 of 35 2 of 3
*Rosa chinensis*	4 of 9^NN^	3 of 4^NN^	0.64			1 of 4	6 of 9
*Cucumis sativus*	6 of 8	2 of 5	0.82			3 of 5	1 of 8
*Prunus dulcis*	10 of 17^NN^	6 of 14^NN^	0.88	1 of 14	1.13	7 of 14	10 of 17
*Carica papaya*	3 of 7	1 of 3	0.42	1 of 3	1.4	1 of 3	1 of 7

**Notes:** The non-functionality ratio refers to the structurally deficient catechol oxidases (COs) *vs.* all COs in a given species. FP means species have low COs non-functionality and high positive selection ratio, whereas NN refers to species that have high COs non-functionality and the high purifying selection ratio.

### Structural-functionality and selective pressure were partially responsible for the species-dependent distribution of COs

COs with any of the six His residues absent might be unable to oxidize phenolic substrates, and were identified as non-functional [31, 42]. We analysed the structural non-functionality ratio, and the selective pressure to understand why COs decreased in some species, while were retained in other species. The results revealed that the structural non-functionality of COs occurred species-dependently, and it happened frequently in some species such as *M. ploymorpha, Oryza sativa Japonica, Nyssa sinensis, C. papaya, Rosa chinensis, Cucumis sativus* and *Prunus dulcis.* Furthermore, more COs were under purifying selection in some of these species (*M. ploymorpha*, *P. dulcis, C. papaya,* and *R. chinensis*) (Table 1; Table S2, see online supplementary material). These negatively selected and structurally non-functional COs might gradually disappear from the genome, and lead to the contraction of COs in some species. By contrast, non-functional COs occurred at lower frequency, and more COs were under positive selection in *C. braunii*, *Azolla filiculoides*, *Cinnamomum micranthum*, and *Nicotiana tabacum* (all COs were structurally functional and under positive selection) (Table 1). Therefore, structural-functionality and selective pressure might be partially responsible for the species-dependent retention or contraction of COs in some species.

### The non-functionality of COs is independent of chloroplast targeting

The ancestral cytosolic variant of heterologous COs might be deleterious to plant growth compared with the modern plastid-localized ones [23, 43]. Evolutionary forces would favor the fixation of the plastid-localized enzyme within the population [23]. Therefore, the correspondence of chloroplast targeting and the species-specific non-functionality of COs was explored. Generally, the chloroplast targeting frequency gradually increased from charophytes, bryophytes, and ferns to seed plants (Table 1), supporting their HGT origin.

However, the chloroplast localization was not necessarily related to non-functionality as detected by Fisher Test (Table S2; see online supplementary material). This could also be revealed by fewer COs in *C. braunii* being targeted to the chloroplasts, but most of them were under positive selection*,* and had functional structures. A larger proportion of COs was predicted to localize in the chloroplasts in seed plants, but the gene family contracted or was absent in some linages. The low chloroplast-targeting ratio of COs in *C. sativus* and *C. papaya* corresponded with their high frequency of structural non-functionality (Table 1). However, only one chloroplast-targeted COs in these two species was also structurally non-functional (Table S2, see online supplementary material). These data indicated a lack of correspondence between chloroplast targeting and COs non-functionality.

## Discussion

Phenols, especially flavonoids, contribute to coping with increased exposure to UVB, drought, and pathogens, and also function in plant growth and reproduction [44]. Phenoloxidases are responsible for the polymerization and formation of various polyphenolic compounds [6]. However, as two major groups of phenoloxidases with overlapping oxidation activity against phenolic substrates, it is unclear how PPOs and LACs evolved and helped plants colonize land. Our analysis showed that TYRs and AAOs were defined as the first-stage phenoloxidases in Chlorophyta (Fig. 7). The COs might have been introduced by HGT from bacteria, expanded in charophytes and bryophytes, and were the second-stage phenoloxidases playing a major role during the early land colonization (Fig. 7). The LACs evolved from the AAOs and prevailed in the vascular plants; they are considered as the third-stage phenoloxidases adapted to more diversified phenol substrates and advantageous to the erect growth of vascular plants (Fig. 7).

### The HGT origin of COs and their irreplaceable roles during land colonization

The PPO proteins might be of ancient origin due to many different HGT events during evolution [23]. The present study provided evidence for the bacterial origin of streptophyte COs, and the ‘core’-Streptophyta COs might be the evolutionary progeny of COs obtained from Nitrospiraceae [26]. The similarity of domain structure, key residues, the 3-D structures, and compositional biases, the gradually increased chloroplast targeting, and the number of genes with introns collectively supported the homology of Streptophyta COs to those of bacteria. This HGT might have happened in the common ancestor of Charales and embryophytes already living in a semi/terrestrial environment.

Several studies identified phenolic compounds in streptophyte algae, such that massive ‘phenolic UV light screens’ found in some charophytes [3], the phenylpropanoid-derived compounds in Zygnematophyceae [45], and mycosporine-like amino acids (MAAs) derived from the shikimate pathway in members of Klebsormidium [46]. These compounds acted as sunscreens to protect these streptophyte algae against UV irradiance [3, 45, 46]. Bi- and tri-flavonoids are common in bryophytes, and moss produce flavonols glycosides, bioflavonoids, and cell wall-bound phenolics in response to UVB exposure [38]. In *M. polymorpha*, mutants with reduced concentration of flavonoids are damaged more easily by UVB [44, 47].

COs emerged and expanded in Klebsormidiales, Charales and bryophytes, with fewer LACs found, except in hornworts. They could have key roles in the metabolism of the phenolics compounds in these groups [16] due to their reported function in the tyrosine, phenylpropanoid and flavonoids biosynthetic pathways [30, 44]. An example is that COs in *M. polymorpha* were involved in the biosynthesis and/or polymerization of auronidin-riccionidin A, the newly reported phenylpropanoid pigments found in several liverwort species, and their main function might be light screening [16, 38, 48, 49]. Therefore, the COs in streptophyte algae and bryophytes might have irreplaceable roles during land colonization.

### The varied subcellular localization and proposed specialized functions of COs

COs should have diversified subcellular localization adapting to a variety of specific functions, such as protection against biotic and abiotic stresses, plant development [50], cell differentiation and death [30], and the UVB screening [16]. Indeed, COs were reported to localize in the chloroplast, vacuole, Golgi apparatus, and mitochondria [24, 51]. The non-chloroplast targeted COs of *K. nitens*, C. braunii and *P. patents*, and the riccionidin A biosynthesis COs in *M. polymorpha* might have been adapted to the metabolism of their proposed phenolic compounds and the UV-B screening function, which might yield more benefits for the land adaption than the proposed deleterious effects to plant growth due to their non-chloroplast targeting [23, 43]. Therefore, most of the non-chloroplast targeted COs are retained in these species.

COs were retained in some species that might have specialized substrates and roles in plant development. For example, COs are specialized as aurone synthase in Asteraceae and Fabaceae [15, 16, 52]. In creosote bush, CO hydroxylates the phenolic compound larreatricin with very limited distribution [53], and oxidizes betalin pigments limited to Caryophyllales [30]. Therefore, the species that specially retained COs might have specialized substrates, and these COs might play irreplaceable roles in the limited number of taxa containing their specialized substrates [52]. The phenylpropanoid pathway produces lineage-specific metabolites [26], and COs might be one of the enzymes regulating metabolite specificity in these routes.

*M. domestica* and *S. tuberosum* were the representative species of angiosperms with conspicuously retained COs (Table 1, Fig. 6). Except for being responsible for the browning reaction in tubers of potato and fruit of apple, COs and specific phenols also play a role in resistance against potato soft rot infection [54], fire blight and grey mold disease of apple [55, 56], the penetration and spread of the pathogen, and wound stress responses [54, 57]. Further work is needed to identify the lineage-specific substrates and functions of COs, especially in the species that have retained COs in abundant quantities.

### Laccases (LACs) are the main phenoloxidases in vascular plants

Even though LACs evolved earlier in streptophyte algae (Zygnemaphyceae) and bryophytes, their number was more than COs only in ferns, and strongly expanded in seed plants, indicating their leading role in the vascular plants. One possible explanation for their domination in vascular plants might be that laccases play a key role in the polymerization of lignin monomers, and thus lignin biosynthesis (structural component essential for all vascular plants) [58, 59]. Therefore, the evolution of laccases might be critical to the erect growth, xylem vessel formation, and structural reinforcement upon pathogen attack of vascular plants [26, 58]. In addition, the shikimic acid, phenylpropane and flavonoids pathways expanded greatly in seed plants (Fig. 6). This expansion inevitably led to the diversification of phenols and flavonoids [59]. The low substrate specificity of laccases facilitates the catalysis and metabolism of diverse phenols [22, 60]. The LACs that catalyse a broad range of substrates might be superior in seed plants with diversified phenols. In species with abundant LACs and lacking COs specialized substrates, or in species with COs undergoing purifying selection and having frequent structural non-functional enzyme center (Table 1), COs might gradually contract or even lose in these linages.

## Conclusion

The COs and LACs are involved differentially in plant transition to land in the process of evolution. COs emerged and expanded in the pioneer land-colonizing ancestor, and might have played an irreplaceable role in the early land colonization. The retention of COs in seed plants was species-dependent, corresponding to the reported limited taxonomic distribution of their specialized substrates. The LACs evolved from the AAOs, became dominant phenoloxidases in the vascular plants, and expanded strongly in seed plants. Their low substrate specificity might be advantageous regarding more diversified phenol substrates, and critical in the lignin biosynthesis supporting the erect growth of vascular plants and the species diversity of seed plants [58, 61]. These findings promote our understanding of the origin and the diversified functions of phenoloxidases during plant evolution and would facilitate the future research into their functions.

## Materials and methods

### Dataset of protein sequences and screening for homologs

The plant proteomes were downloaded from public databases (Table S1, see online supplementary material). All plant proteomes were clustered using OrthoFinder v2.5.4 with DIAMOND for protein alignment to identify orthologous groups of proteins [62], and the clusters containing PPOs (TYRs and COs), LACs (including AAOs), and the shikimate, phenylpropane and flavonoids pathways genes were extracted. Orthologous protein numbers were collected from the Orthogroups. The GeneCount file was verified in the PFAM database, and the members without characteristic PFAM domains were eliminated.

The PPOs, LACs, and AAOs of bacteria, cyanobacteria, chlorophytes, and fungi were obtained from NCBI. The COs proteins of *K. nitens* (GAQ90885) or *C. braunii* (GBG87042) were used as queries to search for the PPOs (TYRs and COs) proteins of bacteria, cyanobacteria, and chlorophytes, by the BLASTP function with an e cutoff value of 10^−5^. The obtained sequences were verified in the PFAM database (http://pfam.xfam.org/), and only the sequences with the tyrosinase, PPO1_DWL and/or the PPO1_KFDV domains were deemed as the bona fide PPOs. The LACs of *S. muscicola* (SM000216S06562) or AAOs of *K. nitens* (GAQ80786.1) were used as queries to search for the LACs and AAOs of bacteria, cyanobacteria, and fungi, and obtained sequences were tested similarly.

### Alignments and phylogenetic analysis

The Pfam domain sequences (Tyrosinase, PPO1_DWL and the PPO1_KFDV) of representative PPOs (GAQ 81141.1 of *Klebsormidium nitens*, PTQ 26780 of *M. polymorpha*, and RZC 46468 of *Papaver somniferum*) were obtained in SMART (http://smart.embl-heidelberg.de/smart/set_mode.cgi? NORMAL = 1). Three domain sequences of each representative PPOs were concatenated into one sequence. Multiple alignments of these PFAM sequences and the homologous PPOs proteins were done using MUSCLE algorithm with default parameters in the MEGA X [63]. Only the amino acids aligned to the PFAM domain sequences were extracted. The PFAM domain sequences (Cu-oxidase_3, Cu-oxidase, and Cu-oxidase_2) of representative LACs and AAOs (PTQ38697 and PTQ45955 of *M. polymorpha*, XP_016713501.2 of *G. hirsutum*) were obtained in a similar way.

The exported fasta file was used to generate alignments using MAFFT version 7.0 [64] (Supplementary fasta files available in https://figshare.com/projects/Structure_of_plant_catechol_oxidases_and_laccases/142893). The maximum likelihood phylogeny was built using IQ-TREE [65] in PhyloSuite [66]. ModelFinder was applied to select the best-fit model using the BIC criterion [67]. The best models were: VT + G4 for PPOs (Fig. 2) and LG + R7 for plant laccase (Fig. 5) (20 000 and 100 000 replicates of ultrafast bootstraps, respectively), LG + G4 + F for Streptophyta catechol oxidases (Fig. 3) and LG + I + G4 + F for laccase+ascorbate oxidase (both with 10 000 replicates of ultrafast bootstraps) (Fig. 4) [68]. Maximum likelihood phylogenies were inferred using IQ-TREE [65, 69], and the Shimodaira-Hasegawa-like approximate likelihood-ratio test (SH-aLRT) [70].

### Functional residues analysis of PPOs, LACs, and AAOs

Functional residues of PPOs were extracted from the complete protein sequences using MUSCLE algorithm [61] according to the reported key amino acids near the substrate-binding pockets of plant COs [12, 15]. To identify the amino acids around the substrate-binding pockets of plant LACs, the three-dimensional structure of LACs in the representative species of chlorophytes, charophytes, bryophytes, ferns, and seed plants was predicted by AlphaFold 2 algorithm with parameters (—db_preset = full_dbs —model_preset = monomer_casp14) [71]. All protein structures were visualized using the PyMOL open-source version (https://github.com/schrodinger/pymol-open-source), and their functional residues around the substrate-binding pockets were identified. The corresponding functional residues of other plant LACs and AAOs were detected by aligning their complete protein sequences to the functional residue identified above [61]. The three-dimensional structures of TYRs and COs were also similarly predicted and visualized.

### Compositional biases and introns analysis

We used Python 3.9.7 with CAI and Bio packages to calculate relative synonymous codon usage (RSCU) to analyse the compositional biases. The bacterial, archaeal and plant plastid codes (genetic code table 11) and the standard code (genetic code table 1) were applied to the sequences of *Nitrospira spp.* and others. The ‘average’ method was chosen for cluster analysis, and dendrogram was generated by Python 3.9.7 with SciPy, Pandas, MatPlotLib, and SciencePlots packages [72]. The gene sequences were downloaded, and their structure was analysed in NCBI and displayed in Gene Structure Display Server Version 2.0 (http://gsds.gao-lab.org).

### Ka/Ks calculation and selection pressure analysis of COs

We identified the duplicated gene pairs within a given species in Table 1 using Blastall, Samtools, and MCScanX software based on their coding sequence alignments with the following criteria: the similarity of aligned regions of two genes >70%, and the coverage >80% [73–75]. The duplicated gene pairs that met these criteria were subsequently designated the non-synonymous (Ka) and synonymous (Ks) substitutions, and the evolutionary constraint (Ka/Ks) was calculated using Ka/Ks_calculator 2.0 software [76]. The duplicated gene pairs with Ka/Ks value >1, <1 and =1 were analysed to represent genes under positive, purifying, and neutral selection, respectively [76].

### The structural functional analysis of COs

The copper ions in active center of COs are coordinated by six conserved histidines (HisA1, HisA2, HisA3, HisB1, HisB2, and HisB3) [27, 41]. COs with any of these His residues absent have structural defective enzymatic centers, and might lose their ancestral function to oxidize phenolic substrates [31, 42]. Therefore, the COs with either His residues lost were identified as non-functional. The complete sequences of Streptophyta COs were aligned by the MUSCLE algorithm with default parameters in the MEGA X [61]. The His residues were searched for, and those with any of the six His residues absent were counted as non-functional COs.

### Prediction of chloroplast localization

The chloroplast targeting of Streptophyta COs was predicted with WoLF PSORT (https://wolfpsort.hgc.jp/) [77]. The COs with the score of >7 were considered as being likely targeted to chloroplast.

### Statistical analysis and graphics

Conserved domains of PPOs were predicted using PfamScan [78] (https://www.ebi.ac.uk/Tools/pfa/pfamscan/). The phylogenetic tree was annotated using iTOL (https://itol.embl.de/) [79], and colored in Adobe Photoshop cs5.1 and Adobe illustrator 2021. The relevance of structural functionality and chloroplast targeting were analyzed by Fisher's exact test in SPSS 26.

## Acknowledgements

This work received financial support from the National Natural Science Foundation in China (Grant No. 32060175 and 32060043), and Fundamental Research Projects of Yunnan Province (2022530401740002). The authors would like to express their gratitude to EditSprings (https://www.editsprings.cn) for the expert linguistic services provided.

## Author contributions

J.L. and S.C. planned and designed the research. J.L. and X.W. collected the data and contributed equally to the manuscript writing. X.W., K.T., J.S., G.C., Z.W., and M.F. analysed the data. G.D., Q.Q., S.S., and S.F. revised the paper. All authors gave final approval for publication.

## Data availability

All PDB files of the predicted three-dimensional structure of catechol oxidases and laccases are available in https://figshare.com/projects/Structure_of_plant_catechol_oxidases_and_laccases/142893. All aligned fasta files used to compute the phylogenetic tree, and Tables S1 and S2 are also available in the database. The nodes with bootstrap support below 80% were collapsed in the phylogenetic tree of Fig. 5, and this collapsed tree was also shown in this database.

## Conflict of interest statement

The authors declare no competing interests.

## Supplementary data

Supplementary data is available at *Horticulture Research* online.

## Supplementary Material

Web_Material_uhad102
