# Supplementary material for: Phenoloxidases: catechol oxidase – the temporary employer and laccase – the rising star of vascular plants
Source: Hortic Res. 2023 May 16;10(7):uhad102. doi: 10.1093/hr/uhad102 (PMC10541563; doi:10.1093/hr/uhad102)
Supplement: Web_Material_uhad102 [file web_material_uhad102.zip › supplementary legends.docx]

**Supplementary Fig. 1**

**Enzymatic center structures of catechol oxidases built by AlphaFold 2**

The conserved sequences of tyrosinase (PF00264.23), PPO_DWL (PF12142.11), and PPO_KFDV (PF12143.11) domain are distinguished by HMMER website (https://www.ebi.ac.uk/Tools/hmmer/search/hmmscan), and are coloured in palegreen, lightblue, and paleyellow, respectively. Residues beyond these conserved sequences are coloured in palecyan. Three histones of CuA and CuB are respectively coloured in red and magenta, gatekeeper residues are coloured in yellow, and the water-keeper residues are coloured in orange. The complete 3-D structure of these proteins are available in https://figshare.com/projects/Structure_of_plant_catechol_oxidases_and_laccases /142893.

**Supplementary Fig. 2**

**Comparation of substrates binding pockets residues between the Chlorophyta** **tyrosinase and the** **catechol oxidases of Streptophyta algae**

**Supplementary Fig. 3**

**The compositional biases of catechol oxidases (COs) represented by relative synonymous codon usage (RSCU) cluster heatmap**

The bacterial, archaeal and plant plastid code (genetic code table 11) and the standard code (genetic code table 1) were applied to the sequences of *Nitrospira spp*., and others, respectively.

**Supplementary Fig. 4**

**Introns analysis of catechol oxidases (COs) displayed in Gene Structure Display Server Version2.0.**

**Supplementary table 1**

The source and information of plant proteomes

**Supplementary table 2**

The detailed information of structural non-functionality, selective pressure, and chloroplast targeting of catechol oxidases
