# Supplementary figures and images for: Phenoloxidases: catechol oxidase – the temporary employer and laccase – the rising star of vascular plants
Source: Hortic Res. 2023 May 16;10(7):uhad102. doi: 10.1093/hr/uhad102 (PMC10541563; doi:10.1093/hr/uhad102)

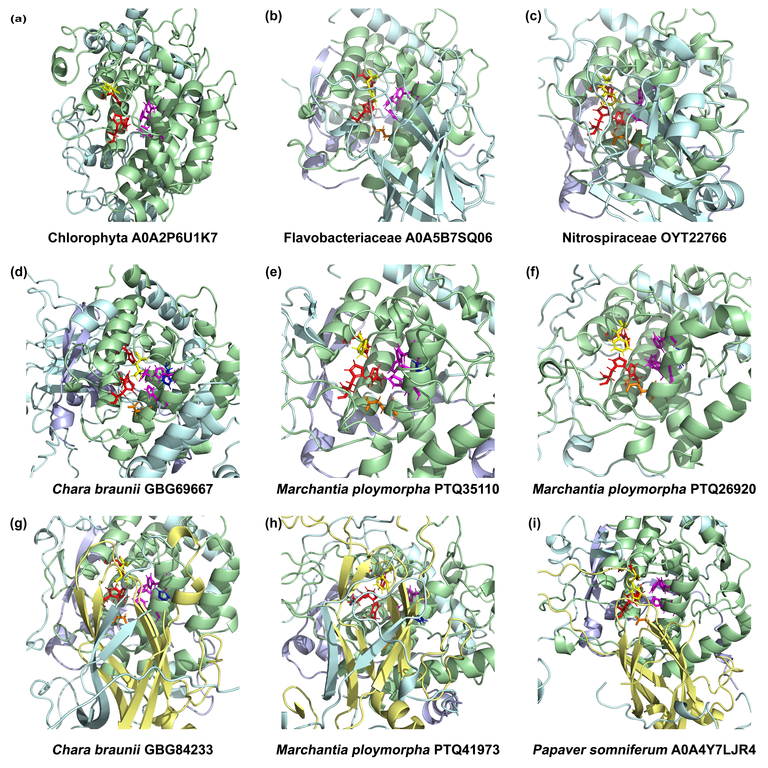

Supplement: Web_Material_uhad102 [file web_material_uhad102.zip › imgonline-com-ua-resize-eAAZ6j67TWp8F.tif]

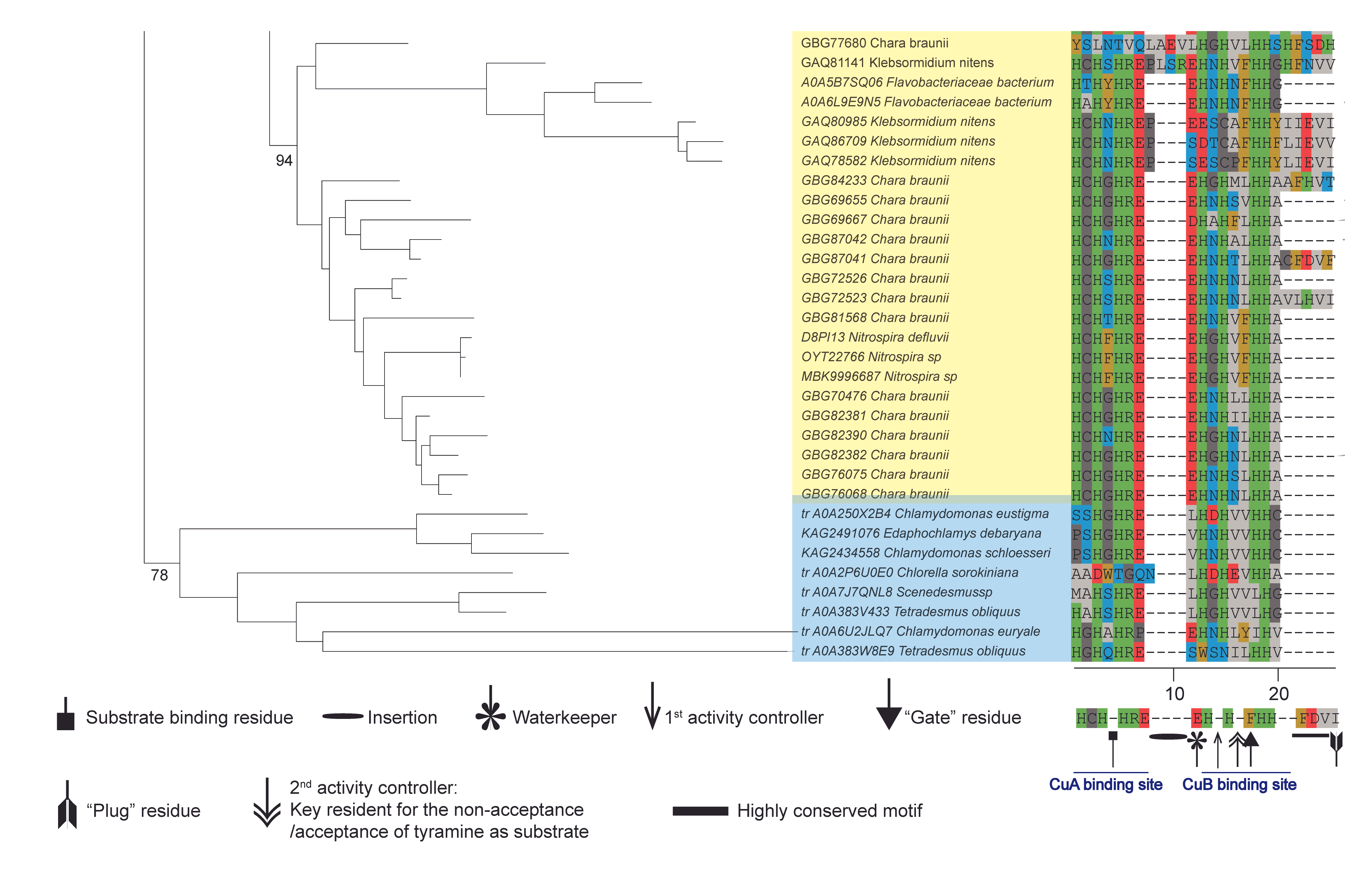

Supplement: Web_Material_uhad102 [file web_material_uhad102.zip › Supplementary Compressed Figure 2.tiff]

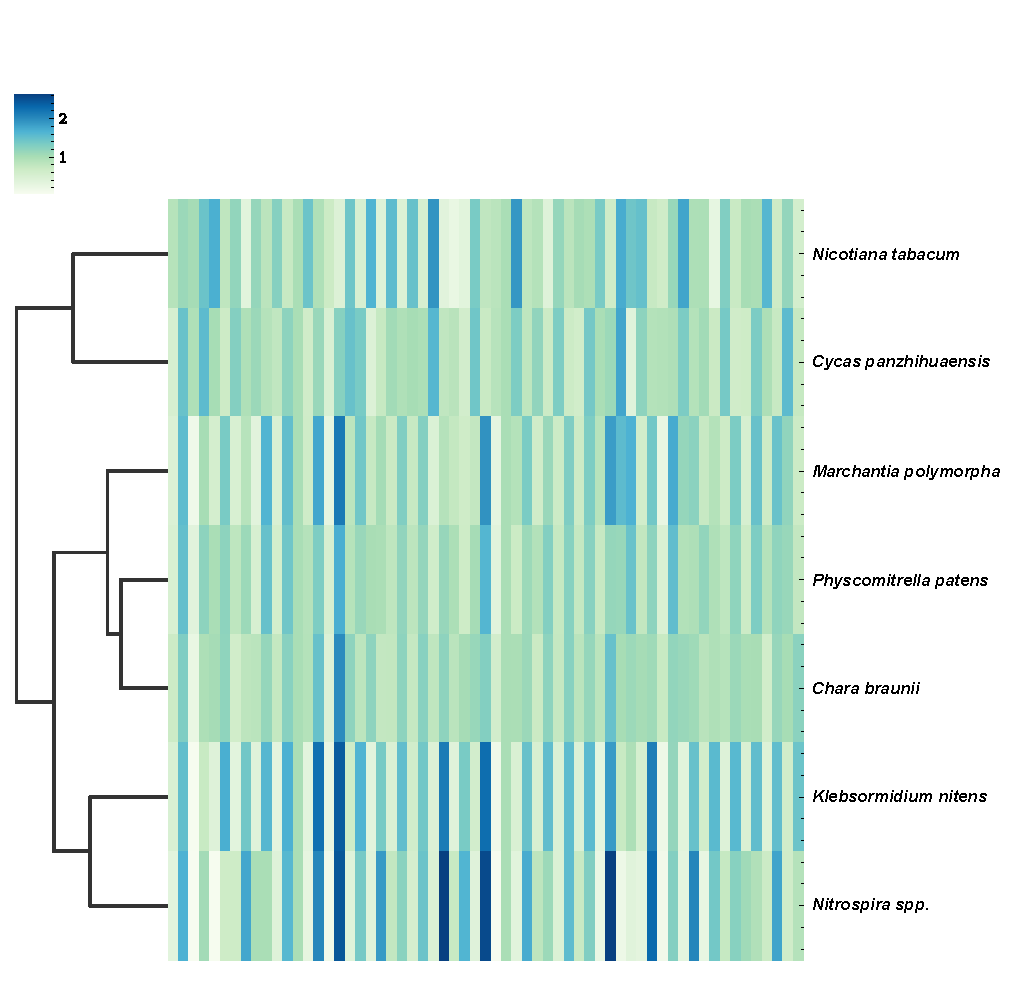

Supplement: Web_Material_uhad102 [file web_material_uhad102.zip › Supplementary Figure 3.tif]

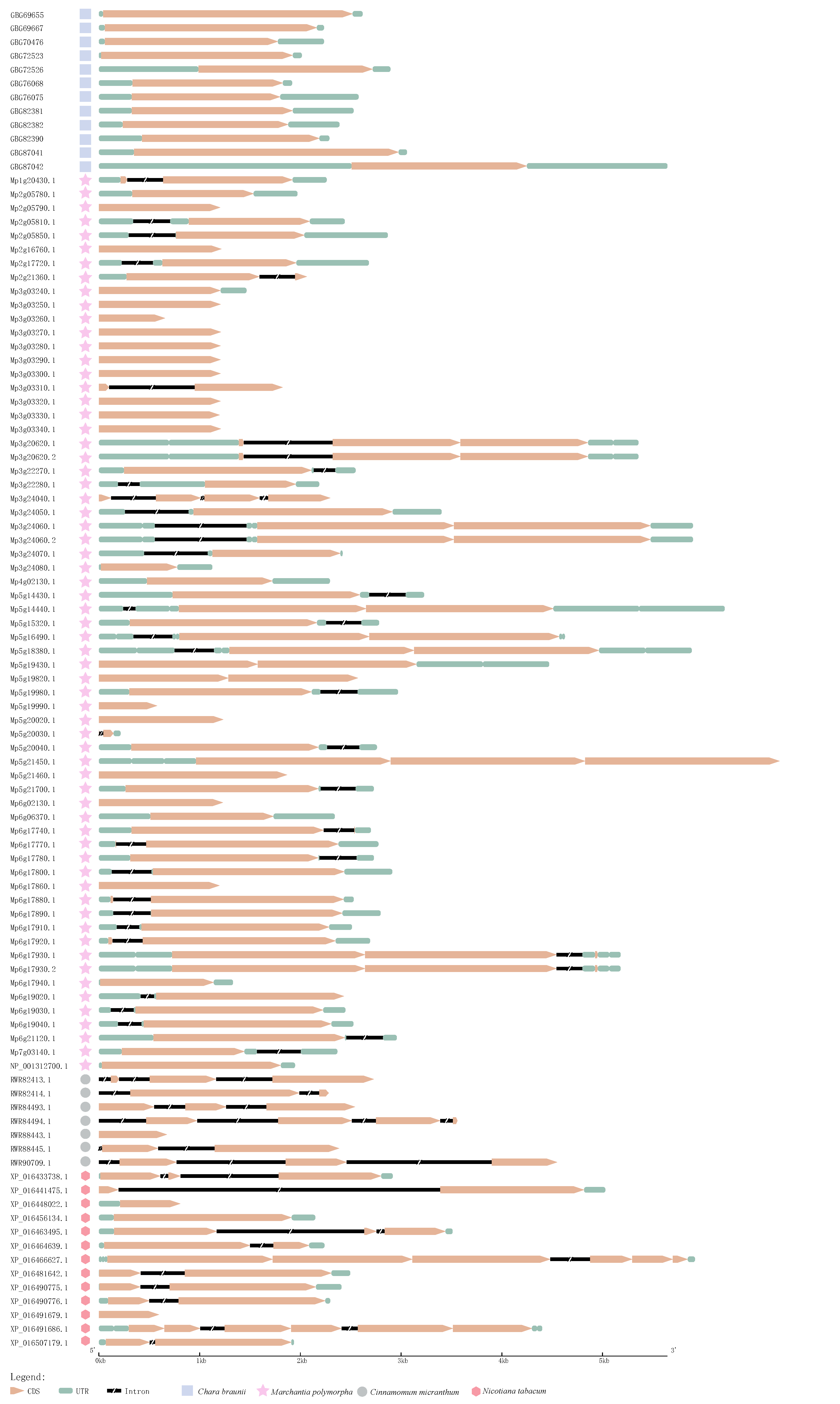

Supplement: Web_Material_uhad102 [file web_material_uhad102.zip › Supplementary Figure. 4.tif]
